# “Decline and uneven recovery from 7 common long-term conditions managed in the Catalan primary care after two pandemic years: an observational retrospective population-based study using primary care electronic health records”

**DOI:** 10.1186/s12875-022-01935-0

**Published:** 2023-01-14

**Authors:** Núria Mora, Francesc Fina, Leonardo Méndez-Boo, Roser Cantenys, Mència Benítez, Nemesio Moreno, Elisabet Balló, Eduardo Hermosilla, Mireia Fàbregas, Carolina Guiriguet, Xavier Cos, Sara Rodoreda, Ariadna Mas, Yolanda Lejardi, Ermengol Coma, Manuel Medina

**Affiliations:** 1 Primary Care Services Information Systems (SISAP), Institut Català de la Salut (ICS), Gran Via de Les Corts Catalanes, 587, 08007 Barcelona, Spain; 2 Equip d’Atenció Primària de Gòtic, Institut Català de la Salut (ICS), Barcelona, Spain; 3 Institut Universitari d’Investigació en Atenció Primària Jordi Gol (IDIAPJGol), Barcelona, Spain; 4 Universitat de Barcelona, Barcelona, Spain; 5 Direcció Assistencial d’Atenció Primària i a la Comunitat, Institut Català de la Salut (ICS), Barcelona, Spain; 6 DAP_Cat Research Group, Gerència Territorial Barcelona Ciutat, Institut Català de la Salut, Barcelona, Spain; 7 Universitat Autònoma de Barcelona, Barcelona, Spain; 8 CIBERDEM, ISCIII , Madrid, Spain

**Keywords:** COVID-19, Electronic health records, Chronic diseases, Primary care, Incidence

## Abstract

**Background:**

The incidence of chronic diseases during the COVID-19 pandemic has drastically been reduced worldwide due to disruptions in healthcare systems. The aim of our study is to analyse the trends in the incidence of 7 commonly managed primary care chronic diseases during the last 2 years of the COVID-19 pandemic in Catalonia.

**Methods:**

We performed an observational retrospective population-based study using data from primary care electronic health records from January 2018 to August 2022 (5.1 million people older than 14 years). We divided the study period into two: a pre-pandemic period (before 14 March 2020) and a pandemic period. We performed a segmented regression analysis of daily incidence rates per 100,000 inhabitants of 7 chronic diseases: type 2 diabetes mellitus (T2DM), asthma, chronic obstructive pulmonary disease (COPD), ischemic heart disease (IHD), heart failure (HF), hypertension and hypercholesterolemia. In addition, we compared annual incidence between pandemic years (2020, 2021 and 2022) and 2019. Associated incidence rate ratios (IRR) were also calculated. Finally, we estimated the number of expected diagnoses during the pandemic period using data from 2019 and we compared it with the observed data.

**Results:**

We analysed 740,820 new chronic diseases’ diagnoses. Daily incidence rates of all 7 chronic diseases were drastically interrupted on 14 March 2020, and a general upward trend was observed during the following months. Reductions in 2020 were around 30% for all conditions except COPD which had greater reductions (IRR: 0.58 [95% CI: 0.57 to 0.6]) and HF with lesser drops (IRR: 0.86 [95% CI: 0.84 to 0.88]). Some of the chronic conditions have returned to pre-pandemic diagnosis levels, except asthma, COPD and IHD. The return to pre-pandemic diagnosis levels compensated for the drops in 2020 for T2DM and HF, but not for hypertension which presented an incomplete recovery. We also observed an excess of hypercholesterolemia diagnoses of 8.5% (95%CI: 1.81% to 16.15%).

**Conclusions:**

Although primary care has recovered the pre-pandemic diagnosis levels for some chronic diseases, there are still missing diagnoses of asthma, COPD and IHD that should be addressed.

**Supplementary Information:**

The online version contains supplementary material available at 10.1186/s12875-022-01935-0.

## Background

The COVID-19 pandemic and the public health measures implemented to contain the spread of the SARS-CoV-2 virus have negatively affected all levels of health systems around the world [1–4]. Besides the direct effects of COVID-19 related illnesses, several studies have described disruption in the healthcare of non-COVID patients which included the suspension of screening programmes or some diagnostic procedures, the reduction of prevention, treatment and control of patients with chronic conditions; and a decline in the number of new non-COVID diagnoses, among others [2, 3, 5–13].

The reduction of non-COVID diagnoses has been described in several countries, for both acute and chronic disease [7]. But unlike infectious diseases [14, 15], the drop of chronic disease diagnoses does not usually imply a reduction of the real incidence, but rather a reduction of detected cases, suggesting the incidence of a large number of undetected conditions, untreated diseases and hence potentially increased long-term morbidity and mortality [2, 3, 6, 7, 16, 17]. For instance, in a study performed in Catalonia central area in 2020, the reductions of new diagnoses accounted for 31%, with greater drops during the lockdown in Spain (March—June 2020) [9]. Moreover, in a previous study from the same year, we found a reduction of around 25% on the incidence of patients with type 2 diabetes mellitus [11].

Although reductions of chronic disease diagnoses have been well described [7, 9], the majority of the studies are focussed on trends from 2020 and early 2021, which raises doubts about the long-term impact of the pandemic in chronic disease diagnoses and its evolution thereafter. For instance, a study performed in Madrid (Spain) observed that, after huge declines of several long-term conditions diagnosed in primary care in 2020, a recovery on the number of diagnoses was observed during 2021 for some diseases, while others remained missing [18]. In this way, a previous study of our research group showed that the number of cancer diagnoses performed in primary care had returned to pre-pandemic levels by the end of 2021, although this did not compensate for the drops in 2020 and, therefore, missing diagnoses during the two previous years were not fully recovered [19]. Despite these studies, we have scarce analyses regarding the evolution of other chronic conditions after 2020; and thus it is uncertain whether these drops in the early days of the pandemic have resulted in prolonged underdiagnoses after two years.

The aim of our study is to analyse the trends of registered incidences of several common chronic diseases managed in the Catalan primary care through the analysis of data from primary care electronic health records (EHR), after more than 2 years since the beginning of the COVID-19 pandemic. In addition, we aim to quantify if expected drops in 2020 were compensated during the second year of the pandemic and if the results differed depending on long-term conditions or sociodemographic characteristics.

## Methods

### Design and data source

We performed an observational retrospective population-based study using data from the primary care electronic health records (EHR) of the *Institut Català de la Salut* (Catalan Institute of Health; or ICS, its Catalan initials). ICS, the main primary care provider in Catalonia (Spain), manages around 75% of all primary care practices in the Catalan public health system and gives coverage to approximately 5.8 million people. Its demographic is highly representative of the population of Catalonia in terms of geographical area, age distribution and gender [20]. All primary care professionals in Catalonia use the same EHR known as ECAP. ECAP is a software system that serves as a repository for structured data on diagnoses, clinical variables, prescription data, laboratory test results, visits, diagnostic requests and referrals to other health care specialties.

### Participants and study period

We included all patients over 14 years of age assigned to a primary care physician (GP) of ICS. In Catalonia, patients younger than this age are visited by paediatricians and are not included in our study. We identify new diagnoses of any of the following long-term conditions registered in the primary care EHR—according to the International Classification of Diseases, 10th Revision, Clinical Modification ICD-10 CM -: type 2 diabetes mellitus (T2DM), asthma, chronic obstructive pulmonary disease (COPD), ischemic heart disease (IHD), heart failure (HF), hypertension or hypercholesterolemia (Table S1 contain the full list of ICD-10 CM codes used in this study). These conditions were selected mostly because they are common chronic diseases managed in primary care for which GP in Catalonia receive reminders and regular feedback within indicators of the Catalan Healthcare Quality Standard [21].

The study period spanned from January 2018 to August 2022. We divided this period into two periods: a pre-pandemic period (from January 2018 to 13 March 2020) and a pandemic period (from 14 March 2020 until the end of the study). The 14^th^ of March 2020 was the first day of the state of alarm and lockdown in Spain [22].

### Variables

The main variable was the number of new chronic disease diagnoses registered in the primary care EHR, which was obtained from the number of individuals diagnosed for the first time with each chronic condition included in our study. Incidence rates per 100,000 inhabitants were calculated by dividing the number of new chronic disease diagnoses by the population size. The population size was extracted monthly from the primary care EHR and it included the number of patients assigned to a GP at the end of each month.

As secondary variables we used age at the time of diagnosis categorised in 5 groups (15–44, 45–59, 60–69, 70–79 and > 79 years), sex (men/women), rurality and socioeconomic status. We assessed the socioeconomic status using the validated deprivation index based on census data (MEDEA deprivation index) constructed by the project Mortality and socio-economic and environmental inequalities in small areas of cities in Spain (MEDEA project) [23] We categorised this index into four groups, where first and fourth groups are the least and the most deprived areas, respectively. Rural areas were categorised separately and were defined as areas with less than 10,000 inhabitants and a population density lower than 150 inhabitants/km^2^.

### Statistical analysis

We analysed daily incidence rates of each chronic disease from January 2018 to August 2022 using a segmented regression analysis approach, expressed as follows:

$$Y \sim {\upbeta }_{0} + {\upbeta }_{\mathrm{time}} * time + {\upbeta }_{\mathrm{pandemic}} * pandemic + {\upbeta }_{{\mathrm{time}}_{-}\mathrm{covid}} * tim{e}_{-}covid + error$$

where:

*Y* is the 7-day moving average daily incidence.

*Time* is the number of days between the beginning of the study period and the date of diagnosis.

*Pandemic* is a dummy that takes 1 when *Time* is after 14 March 2020 and 0 otherwise.

*Time_covid* is the number of days since 14 March 2020.

ꞵ is the coefficient for the indicated variable.

The slope associated with the pre-pandemic period is estimated by ꞵ_time_, while the slope associated with the pandemic period is estimated by ꞵ_time_ + ꞵ_time_covid_. ꞵ_pandemic_ estimates the effect of the pandemic period on the daily incidence.

In order to calculate the difference of diagnoses between pandemic years and pre-pandemic years, we compared the accumulated monthly incidence for each year; and we calculated the annual incidence rate for each year. Incidence rate ratios (IRRs) with the 95% confidence interval (95%CI) were used to compare the incidence in the years 2020, 2021 and 2022 to the incidence in 2019. For the comparison between 2022 and 2019, we used 2019 data from the same months of 2022 (January—August) as baseline.

Finally, we considered that there was a “diagnostic compensation” when the overall diagnoses observed of each disease during the pandemic period (from 14 March 2020 to 31 August 2022) was statistically similar to the expected diagnoses. To do that, we calculated the expected diagnoses from the mean daily incidence of 2019, we multiplied this value by the number of days and the population of the pandemic period and, then, we divided it by 100,000. We estimated the expected value and its 95%CI. We also calculated the difference between expected and observed diagnoses. Whenever the 95%CI of the difference between expected and observed crossed 0 it was considered to have achieved diagnostic compensation, while an excess of diagnoses was determined when the 95%CI yielded over 0, and no diagnostic compensation when it remained under 0.

The significance level of all statistical tests is alpha = 0.05.

All analyses were conducted using R, version 3.5.1 [24]

## Results

The baseline characteristics of our population are presented in Table 1. The population over 14 years of age slightly increased since 2018 while other characteristics remained stable during the study period in terms of age, sex and socioeconomic status distribution.

**Table 1:** Baseline characteristics of the study population by year (2018—2022)

	**2018**	**2019**	**2020**	**2021**	**2022**
Total	4,852,961 (100%)	4,957,155 (100%)	4,991,716 (100%)	5,053,670 (100%)	5,108,878 (100%)
Age					
15–44	2,183,988 (45%)	2,209,398 (44.57%)	2,216,414 (44.4%)	2,204,497 (43.62%)	2,218,929 (43.43%)
45–59	1,267,021 (26.11%)	1,306,846 (26.36%)	1,334,878 (26.74%)	1,370,374 (27.12%)	1,392,801 (27.26%)
60–69	607,066 (12.51%)	622,656 (12.56%)	635,927 (12.74%)	651,006 (12.88%)	660,190 (12.92%)
70–79	450,636 (9.29%)	479,562 (9.67%)	480,311 (9.62%)	489,048 (9.68%)	499,086 (9.77%)
> 79 years	346,465 (7.14%)	346,589 (6.99%)	346,467 (6.94%)	346,519 (6.86%)	346,001 (6.77%)
Sex					
Men	2,370,318 (48.84%)	2,421,966 (48.86%)	2,440,227 (48.89%)	2,475,074 (48.98%)	2,499,848 (48.93%)
Women	2,482,643 (51.16%)	2,535,189 (51.14%)	2,551,489 (51.11%)	2,578,991 (51.03%)	2,609,701 (51.08%)
Socioeconomic status					
Rural	1,159,868 (23.9%)	1,173,457 (23.67%)	1,187,011 (23.78%)	1,207,685 (23.9%)	1,222,434 (23.93%)
1st Q (least deprived)	1,080,159 (22.26%)	1,093,771 (22.06%)	1,100,788 (22.05%)	1,115,034 (22.06%)	1,128,103 (22.08%)
2nd Q	753,137 (15.52%)	763,990 (15.41%)	769,916 (15.42%)	778,361 (15.4%)	788,254 (15.43%)
3rd Q	991,232 (20.43%)	1,018,627 (20.55%)	1,024,027 (20.51%)	1,034,914 (20.48%)	1,045,552 (20.47%)
4th Q (most deprived)	868,565 (17.9%)	907,368 (18.3%)	913,508 (18.3%)	918,455 (18.17%)	927,458 (18.15%)

Overall, 740,820 new chronic diseases’ diagnoses were registered in the Catalan primary care EHR: 224,937 hypertension diagnosis, 150,214 hypercholesterolemia, 133,540 T2DM, 65,842 asthma, 62,313 HF, 55,762 COPD and 48,212 IHD. The annual number of chronic diseases were similar in 2018 and 2019 (with the exception of hypercholesterolemia, which slightly increased in 2019). However, in 2020 all diagnoses abruptly dropped. Mean age and percentage of women were similar across all years within each chronic condition but the percentage of rural areas decreased in 2020, 2021 and 2022 in some diagnoses (Supplementary Table S2 and Supplementary Figure S1).

**Fig. 1:**
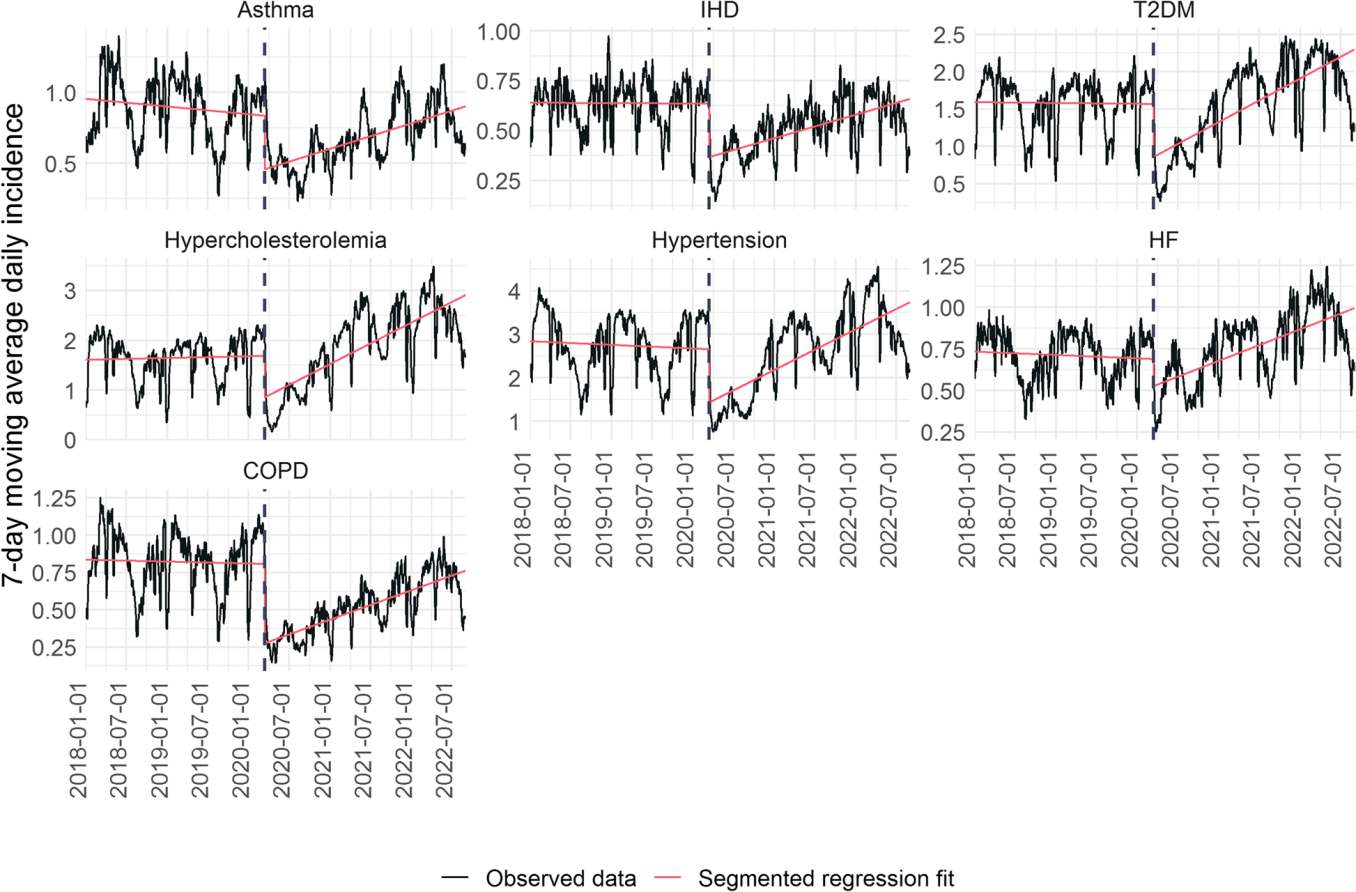
7-day moving average rates of daily chronic diseases since 2018 in Catalonia

Figure 1 shows the results of the segmented regression analysis. The trends of daily rates for all diseases were drastically interrupted on 14 March 2020 (the first day of the lockdown in Spain). During the subsequent months, we observed a general upward trend, although in some conditions the incidence at the end of the study period was still below the pre-pandemic level. Supplementary Table S3 shows the coefficients of the models for each disease.

**Fig. 2:**
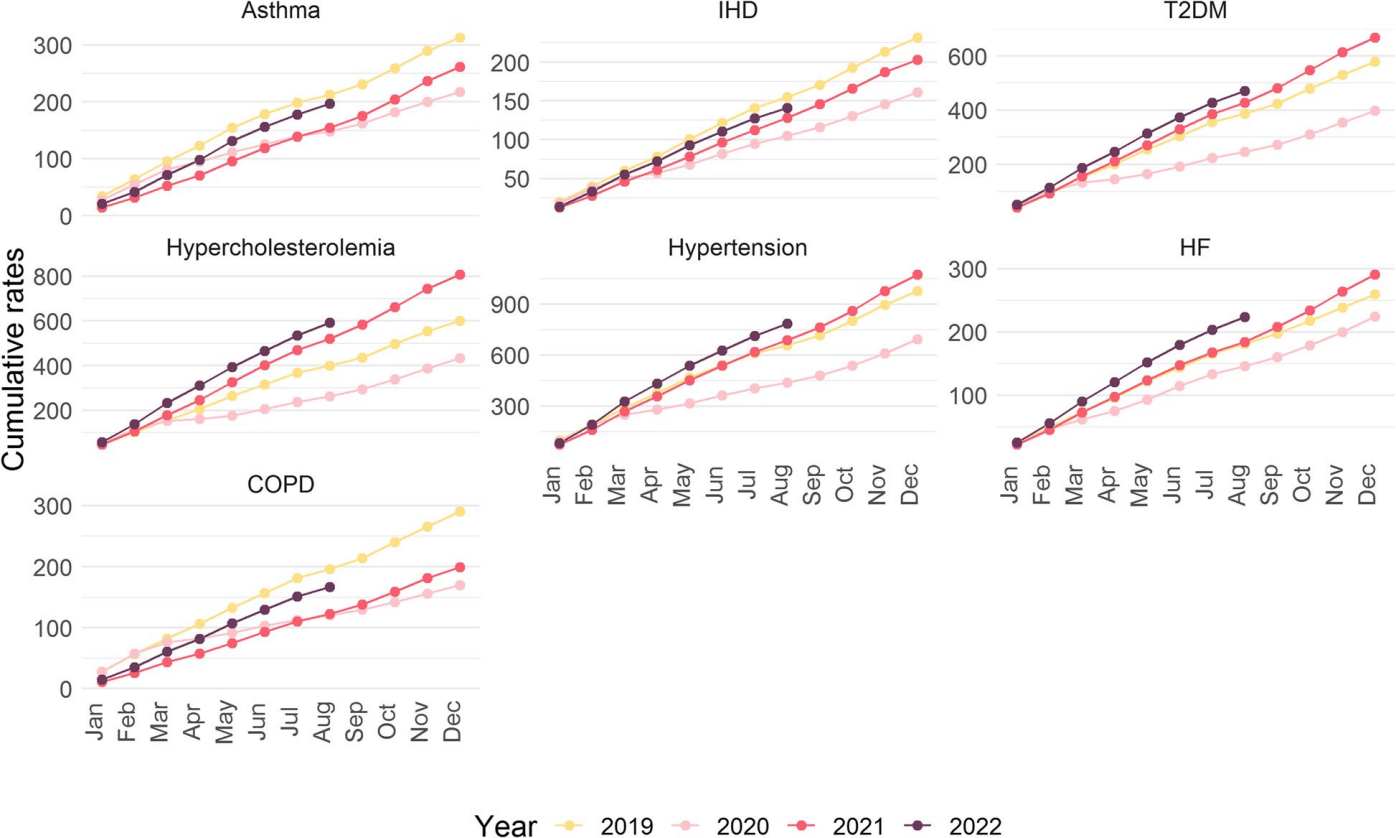
Cumulative rates of monthly chronic diseases registered by year in Catalonia (2019–2022)

Figure 2 shows the monthly-accumulated rates by year of each chronic disease. We observed that the incidence in 2020 decreased for all diseases around March—April 2020. Nevertheless, monthly rates in 2021 and 2022 were hovering at diagnosis level of 2019 or were higher for T2DM, hypertension, HF and hypercholesterolemia. On the other hand, rates of asthma, COPD and IHD remained below 2019 values.

Compared to 2019, the IRR associated with the reductions in 2020 are presented in Table 2 and Figure Supplementary S2. We observed reductions around 30% in asthma, IHD, T2DM, hypertension and hypercholesterolemia; a greater reduction of 42% (IRR: 0.58 [95% CI: 0.57 to 0.6]) in COPD rates and lesser drops in HF (IRR: 0.86 [95% CI: 0.84 to 0.88]). In contrast, in 2021 and 2022, T2DM, hypercholesterolemia, hypertension and HF showed increases in the recorded incidence, with IRR significantly > 1 (Table 2). For instance, in 2021 and 2022, the registered incidence of T2DM increased by 16% and 24%, respectively; and by 34% and 49% in the case of hypercholesterolemia. Conversely, in asthma, COPD and IHD we still observed reductions during 2021 and 2022 although smaller than those from 2020. In particular, COPD presented reductions of 42%, 31% and 15% in 2020, 2021 and 2022, respectively, while asthma diagnoses decreased by 31%, 16% and 7% in the same years.

**Table 2:** Annual rates per 100,000 inhabitants of chronic diseases and incidence rate ratios (IRR) compared to 2019

Chronic disease	Year	Rate × 100,000 2019	Rate × 100,000	IRR	95% CI
Asthma	2020	313.3	216.92	0.69	[0.68—0.71]
	2021	313.3	262.01	0.84	[0.82—0.86]
	2022^$^	211.55	197.34	0.93	[0.91—0.96]
Chronic obstructive pulmonary disease (COPD)	2020	290.05	169.06	0.58	[0.57—0.6]
	2021	290.05	199.4	0.69	[0.67—0.71]
	2022^$^	195.15	166.51	0.85	[0.83—0.88]
Heart failure (HF)	2020	259.6	223.63	0.86	[0.84—0.88]
	2021	259.6	290.9	1.12	[1.09—1.15]
	2022^$^	180.35	223.85	1.24	[1.21—1.28]
Hypercholesterolemia	2020	600.61	432.72	0.72	[0.71—0.73]
	2021	600.61	807.45	1.34	[1.32—1.36]
	2022^$^	397.37	591.75	1.49	[1.46—1.52]
Hypertension	2020	977.29	690.78	0.71	[0.7—0.72]
	2021	977.29	1074.13	1.1	[1.09—1.11]
	2022^$^	653.84	784.58	1.2	[1.18—1.22]
Ischemic heart disease (IHD)	2020	231.99	160.97	0.69	[0.67—0.71]
	2021	231.99	203.46	0.88	[0.85—0.9]
	2022^$^	154.3	141.26	0.92	[0.89—0.95]
Type 2 diabetes mellitus (T2DM)	2020	579.06	396.86	0.69	[0.67—0.7]
	2021	579.06	669.04	1.16	[1.14—1.17]
	2022^$^	386.23	470.85	1.22	[1.2—1.24]

^$^ Data until August

**Fig. 3:**
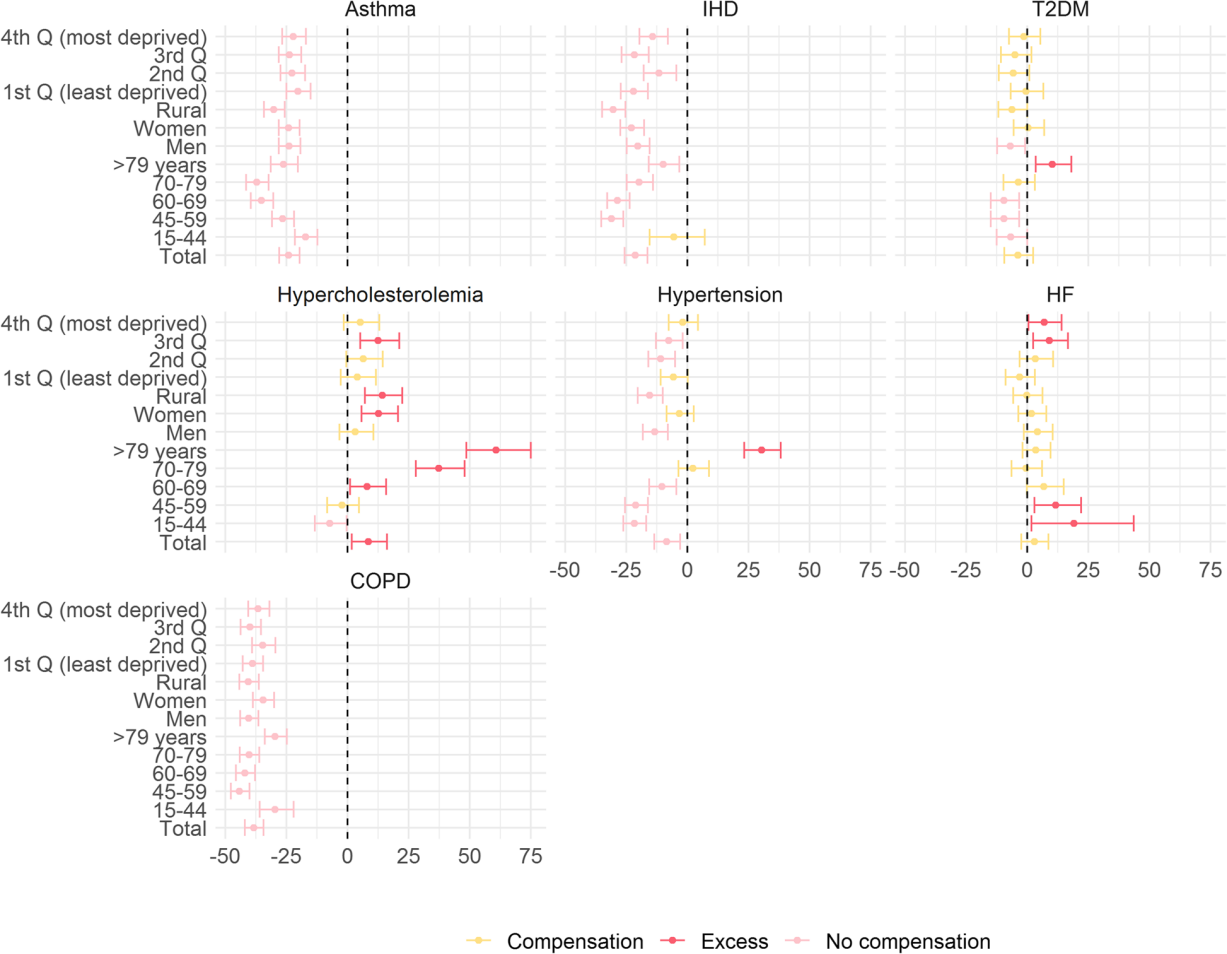
Percentage of difference between observed and expected chronic diagnoses during the pandemic period in Catalonia. Total and by age groups, sex and socioeconomic status

When we compared the observed diagnoses registered during the pandemic period (from 14 March 2020 until 31 August 2022) with the expected ones in order to determine if the increase in some diagnoses in 2021 and 2022 compensated for the drops, we observed, that after more than two years since the beginning of the pandemic, there was still a reduction of -24% (IC95%: -27.94% to -19.68%) in asthma diagnoses, a reduction of -38.39% (IC95%: -41.94% to -34.37%) in COPD diagnoses, a reduction of -21.25% (95%CI: -25.6% to -16.37%) in IHD and a reduction of -8.53% (IC95%: -13.5% to -2.95%) in hypertension. We estimated that the overall reduction during the whole pandemic period accounted for 14,644, 9,719, 6,264 and 10,926 fewer COPD, asthma, IHD and hypertension diagnoses, respectively. Conversely, a 8.51% (95% CI: 1.81 to 16.15%) excess of registered diagnoses was observed in hypercholesterolemia when compared to the expected. Finally, for T2DM and HF we observed a statistically non-significant difference of -3.81% (95%CI: -9.36% to 2.46%) and 2.88% (95% CI: -2.39% to 8.76%) respectively (Fig. 3 and Supplementary Table S4).

In general, similar findings were observed when we stratified data by age groups, sex and socioeconomic status (Fig. 3 and Supplementary Table S4). However, some differences were observed in more advanced age groups with an excess of T2DM and hypercholesterolemia diagnoses; and also with an increase of registered diagnoses of HF in more deprived areas and younger people. No difference were observed regarding sex.

**Fig. 4:**
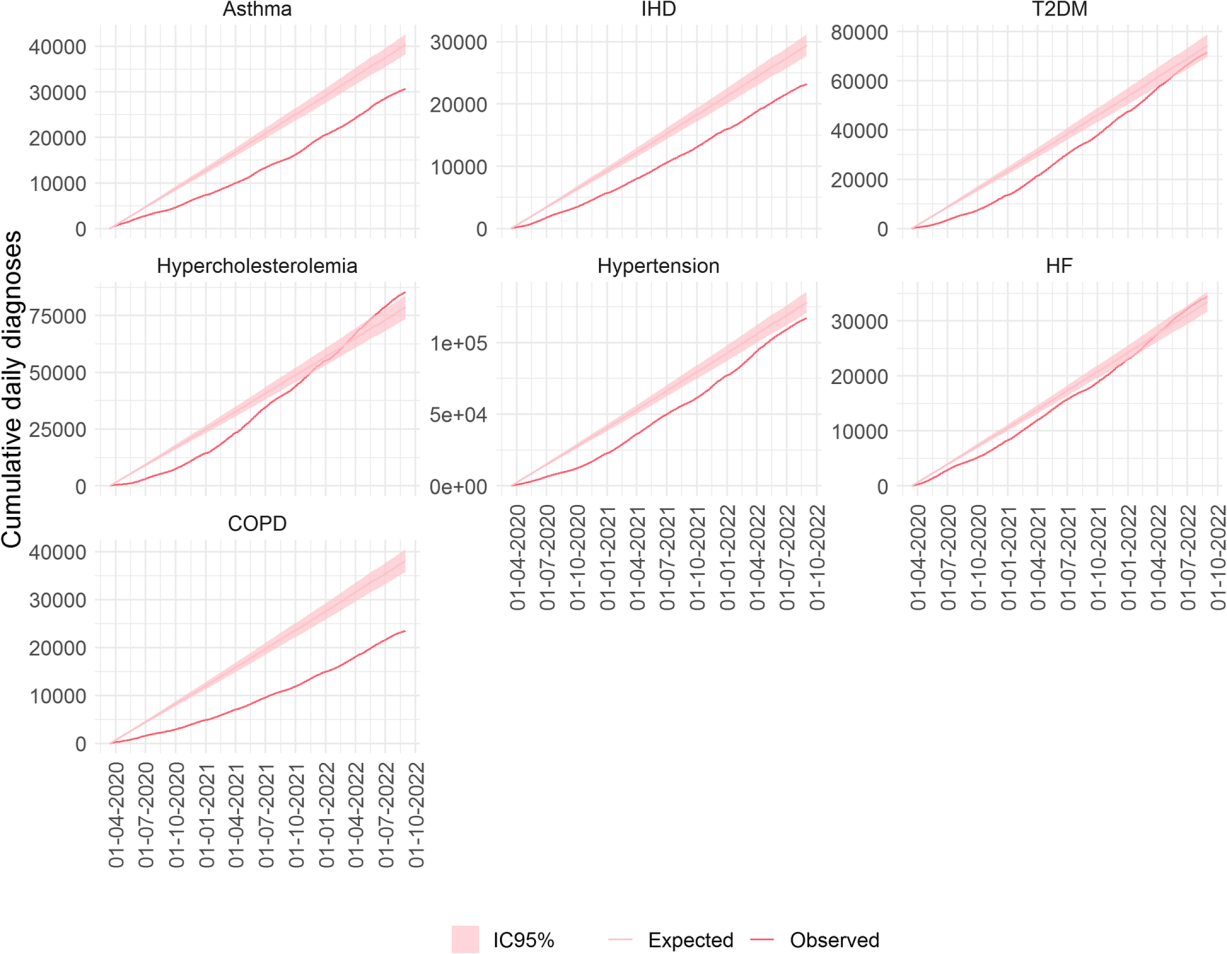
Daily cumulative observed and expected (with 95%CI) new chronic disease diagnoses during the pandemic period (14 March 2020 to 31 August 2022) in Catalonia

Finally, we estimated the time frame when the compensation for T2DM, HF and hypercholesterolemia occurred by comparing the daily cumulative observed diagnoses during the pandemic period to the daily cumulative expected diagnoses. Compensation for hypercholesterolemia took place at the beginning of November 2021, for HF in mid-December 2021 and for T2DM at the end of April 2022. Excess of hypercholesterolemia diagnoses was observed at the end of May 2022 (Fig. 4 and Supplementary Table S5).

## Discussion

We found important reductions ranging between -14% and -42% in recorded new diagnoses of the main long-term conditions managed in primary care during 2020, but an upward increase in some of them in 2021 and the first eight months of 2022. This situation led to a return to pre-pandemic diagnosis levels (or higher) for some chronic diseases except asthma, COPD and IHD, with incidence rates still below those of 2019. The return to pre-pandemic diagnosis levels compensated for the drops in 2020 regarding T2DM and HF, but not for hypertension, which presented an incomplete recovery. Furthermore, we identified an excess of registered diagnoses of hypercholesterolemia (around 8%) that should raise awareness of a possible overdiagnosis or an increase in the number of clinical analyses due to worsening population health.

Our findings for 2020 are aligned with those found in other studies. Several articles have described substantial drops in the incidence of multiple non-communicable diseases. Reductions were observed both in new diagnoses performed during 2020 and new prescribing associated medications [7, 9, 11, 25]. However, few studies have analysed such prolonged periods as the one presented in this study. For instance, an observational study conducted in Madrid (Spain) found that some of the chronic diseases analysed had returned to expected incidence rates, but without compensation [18]. However, the study period only comprised data spanning as far as mid-2021, while some diagnosis compensation found in our study started at the end of 2021. The Madrid study also found a low level of diagnosis for asthma and COPD, just like the present study. Finally, a Belgium study reported reductions of most chronic incidence rates during the first COVID-19 wave but a return to pre-pandemic levels at the end of 2020 and beginning of 2021, also without compensation as in the Spanish article[7].

The return to pre-pandemic levels of diagnosis for most of the chronic diseases analysed in our article points to a recovery of primary care activity. In addition, the compensation of the missing diagnoses for T2DM, hypercholesterolemia and HF, suggests a great effort of primary care professionals in order to reduce the harms of the backlogs that the first year of the COVID-19 pandemic caused in several non-COVID conditions. However, the compensation observed is still incomplete in some conditions, although it is possible that some diseases (such as hypertension) will achieve the expected values in the months to come, since the rates of diagnoses registered in 2021 and 2022 are significantly higher than those of the pre-pandemic threshold. In addition, the fact that two or three face-to-face visits are required for the diagnosis of hypertension (unlike hypercholesterolemia and diabetes, which only require a blood test, the request for and result of which can be telematic), may also explain the lower diagnostic recovery from hypertension, since many of the visits continued to be non-face-to-face.

The persisting low level of asthma and COPD diagnoses is very concerning as patients with undiagnosed conditions are unlikely to be receiving systematic monitoring and management, which could lead to increased morbidity and future negative health outcomes. Reduction in asthma exacerbations during COVID-19 was detected and described in other works [6]. Although the continuing low rates of asthma observed in our study could mark a true incidence drop, the most likely explanation for this low number of registered asthma and COPD diagnosis may be the disruptions of diagnostic procedures, enforced by pandemic guidelines that recommended postponing or reducing some procedures that might increase respiratory transmission, such as spirometry [3, 18]. Further research should be performed to confirm these findings and to identify possible explanations; and appropriate actions should be taken to revert this situation. Regarding the low levels of IHD new diagnoses, some studies have observed reductions in admissions for stroke or heart attack treatments during the pandemic and therefore they suggest that part of the reduction in prevalence and incidence of some chronic diseases could be related to the harvesting effect due to the high impact of the first COVID-19 wave in terms of mortality [26]. However, a study performed in Catalonia in 2020 estimated a low impact of this effect [9]. Conversely, a UK study found a possible trend towards increased strokes after a year from the beginning of the COVID-19 pandemic [27]. It is crucial to establish the reasons behind these still remaining backlogs in future research.

The 8% excess of hypercholesterolemia diagnoses during the pandemic period should also be analysed in depth in future research. Some studies observed an increase of dyslipidemia and obesity during the pandemic, probably related to the decrease of physical activity during lockdowns, remote working or changes in dietary habits [6]. If the reason for this excess of hypercholesterolemia diagnoses is related to worsening population health, we also should expect excess in T2DM and other chronic conditions in the months to come. In this sense, then, it is possible that the compensation of T2DM diagnoses observed was actually a consequence of a greater real hidden incidence and therefore we would still be missing some diagnoses. It is crucial to unravel the causes associated with these increases in future research. However, as in asthma and COPD, it is unlikely that all the observed excess is merely a consequence of a real increment in the incidence of this condition, but rather a possible overdiagnosis that should be taken into consideration and confirmed. In addition, the most recent vascular risk guidelines favour a pharmacological treatment of dyslipidemia, by proposing very low LDL control targets, which is why the diagnosis of hypercholesterolemia is also favoured [28, 29].

Our study has some limitations. First, as our analysis is based on rates, major changes in the population pyramid could limit our findings. However, the population in Catalonia has remained stable in terms of age, sex and socioeconomic characteristics during the study period, as showed in the Results section. Second, the use of primary care EHR could imply a potential lack of hospital diagnoses and a potential diagnoses inaccuracy as rely on routinely collected data. However, Catalan primary care EHR has been previously validated for research, the chronic diseases chosen for the study are usually diagnosed in primary care and, in Catalonia, there have been some healthcare quality indicators calculated with this data for more than 15 years [21, 30, 31]. Third, the lack of more pre-pandemic years to compare could hide possible previous trends. Finally, some alterations in patients’ healthcare seeking behaviour during the COVID19 pandemic could affect the observed trends, although this effect is unlikely to hold throughout 2022.

Despite the limitations, our study also has strengths. Several studies have used the Catalan primary care EHR data to perform useful research in real-world conditions [8, 11, 19, 23, 30–32]. Moreover, our study period lasted for more than two pandemic years and thus it stretches the time frame from the majority of the literature available to date. This long study period allows us to observe not only the decrease of non-COVID conditions but also the recovery and the remaining backlogs and areas to focus on during the following months. In addition, our study includes 7 common long-term conditions managed by primary care professionals that are also included in several quality of care indicators calculated since 2006 and used to monitor these chronic diseases with permanent feedback to GPs [21]. This ensures that in general the coding quality is high. Finally, as ICS manages about three in every four practices in Catalonia, our results may be generalizable and our methods could be introduced in other settings that also use EHR.

## Conclusions

Our work included long-term data showing different trends of chronic disease, with dramatic reductions at the early stages of the pandemic for all 7 chronic diseases analysed here and subsequent recovery of some of them. While in some cases registered diagnoses had been recovered and compensated for the initial drops that took place in 2020 (showing that primary care quickly adapted and resumed pre-pandemic activity), there is still a shortfall in incidence rates of some conditions that should alert policy makers and public health authorities. Some interventions should be carried out to revert this situation, especially changes in COVID-19 guidelines that are no longer justified considering the current epidemiological situation. In addition, more resources are required in primary care to tackle observed and anticipated workload as part of disease recovery and compensation; and specific strategic planning at the level of primary care organisations may be required. Finally, periodic health data about incidence rates of chronic diseases is needed worldwide to track progress in the recovery of missed diagnoses by strengthening clinical information systems and using EHR to perform this continuing disease surveillance.

## Supplementary Information

**Additional file 1:** **Table S1.** ICD-10 CM codes used to identify chronic diseases in the Catalan primary care electronic health records. **Table S2.** Characteristics of the patients with chronic disease diagnoses by year. **Figure S1.** Age distribution of chronic diseases diagnoses during the study period. **Table S3.** Coefficients of the segmented regression model. **Figure S2.** Incidence rate ratios (IRR) by year. Relative to year 2019. **Table S4.** Observed and expected diagnoses during the pandemic period (from 14 March to 31 August 2022). **Table S5.** Date of compensation and excess of diagnoses for each chronic disease. Total and stratified by age groups, sex and socioeconomic status.

## Data Availability

The datasets used during the current study are available upon reasonable request to the corresponding author.

## Abbreviations

95%CI: 95% Confidence interval
COPD: Chronic obstructive pulmonary disease
GP: Primary care physician
EHR: Electronic health records
HF: Heart failure
IRR: Incidence rate ratio
ICS: Institut Català de la Salut
IHD: Ischemic heart disease
T2DM: Type 2 diabetes mellitus
