# Supplementary material for: “Decline and uneven recovery from 7 common long-term conditions managed in the Catalan primary care after two pandemic years: an observational retrospective population-based study using primary care electronic health records”
Source: BMC Prim Care. 2023 Jan 14;24:9. doi: 10.1186/s12875-022-01935-0 (PMC9840158; doi:10.1186/s12875-022-01935-0)
Supplement: Supplementary file 1 — Additional file 1: Table S1. ICD-10 CM codes used to identify chronic diseases in the Catalan primary care electronic health records. Table S2. Characteristics of the patients with chronic disease diagnoses by year. Figure S1. Age distribution of chronic diseases diagnoses during the study period. Table S3. Coefficients of the segmented regression model. Figure S2. Incidence rate ratios (IRR) by year. Relative to year 2019. Table S4. Observed and expected diagnoses during the pandemic period (from 14 March to 31 August 2022). Table S5. Date of compensation and excess of diagnoses for each chronic disease. Total and stratified by age groups, sex and socioeconomic status. [file 12875_2022_1935_MOESM1_ESM.docx]

Supplementary material

Contents

[Table S1. ICD-10 CM codes used to identify chronic diseases in the Catalan primary care electronic health records 2](#_Toc124422158)

[Table S2. Characteristics of the patients with chronic disease diagnoses by year. 3](#_Toc124422159)

[Figure S1. Age distribution of chronic diseases diagnoses during the study period 7](#_Toc124422160)

[Table S3. Coefficients of the segmented regression model 8](#_Toc124422161)

[Figure S2 Incidence rate ratios (IRR) by year. Relative to year 2019 10](#_Toc124422162)

[Table S4. Observed and expected diagnoses during the pandemic period (from 14 March to 31 August 2022) 11](#_Toc124422163)

[Table S5. Date of compensation and excess of diagnoses for each chronic disease. Total and stratified by age groups, sex and socioeconomic status 18](#_Toc124422164)

### Table S1. ICD-10 CM codes used to identify chronic diseases in the Catalan primary care electronic health records

| **Disease** | **ICD-10 CM codes** |
| --- | --- |
| Hypertension | I10, I11.0, I11.90, I12.0, I12.9, I13.0, I13.10, I13.2, I15.0, I15.1, I15.2, I15.8, I15.9, I15.1, I15.11, I15.12, I15.13 |
| Type 2 diabetes mellitus | E11, E11.00, E11.01, E11.10, E11.11, E11.21, E11.22, E11.29, E11.311, E11.319, E11.3292, E11.3293, E11.3312, E11.3393, E11.3513, E11.3519, E11.3553, E11.36, E11.39, E11.40, E11.41, E11.42, E11.43, E11.44,  E11.49, E11.51, E11.52, E11.59, E11.610, E11.618, E11.620, E11.621, E11.622, E11.628, E11.630, E11.638, E11.641, E11.649, E11.65, E11.69, E11.8, E11.9, E13.00, E13.10, E13.11, E13.2, E13.21, E13.29, E13.311, E13.319,  E13.3311, E13.3411, E13.3551, E13.3553, E13.39, E13.40, E13.41, E13.42, E13.49, E13.52, E13.59, E13.621, E13.622, E13.641, E13.65, E13.69, E13.8, E13.9 |
| Hypercholesterolemia | E78, E78.0, E78.00, E78.2, E78.4, E78.5, E78.89, E78.9 |
| Ischemic heart disease | I20, I20.0,I20.8, I20.9, I24.0, I24.1, I24.8, I24.9, I25, I25.10, I25.2, I25.41, I25.5, I25.6, I25.89, I25.9, I70.90 |
| Heart failure | I11.0, I13.0, I13.2, I50, I50.1, I50.20, I50.22, I50.30, I50.32, I50.89, I50.9 |
| Chronic obstructive pulmonary disease (COPD) | J43, J43.0, J43.1, J43.2, J43.8, J43.9, J44, J44.0, J44.1, J44.9 |
| Asthma | J45, J45.20, J45.21, J45.22, J45.30, J45.40, J45.50, J45.901, J45.902, J45.909, J45.998 |

### Table S2. Characteristics of the patients with chronic disease diagnoses by year.

*****Number of diagnoses in 2022 accounted for only 6 months (until June)

| **Chronic disease** | **Year*** | **Total** | **Mean Age (SD)** | **Women (%)** | **Men (%)** | **Rural (%)** | **1st Q (least deprived) (%)** | **2nd Q (%)** | **3rd Q (%)** | **4th Q (most deprived) (%)** |
| --- | --- | --- | --- | --- | --- | --- | --- | --- | --- | --- |
| Asthma | 2018 | 16,160 | 47.47 (19.51) | 10,035 (62.1%) | 6,125 (37.9%) | 3,996 (24.73%) | 3,367 (20.84%) | 2,302 (14.25%) | 3,279 (20.29%) | 3,216 (19.9%) |
|  | 2019 | 15,531 | 46.84 (19.16) | 9,515 (61.26%) | 6,016 (38.74%) | 3,615 (23.28%) | 3,344 (21.53%) | 2,322 (14.95%) | 3,130 (20.15%) | 3,120 (20.09%) |
|  | 2020 | 10,828 | 44.59 (18.21) | 6,618 (61.12%) | 4,210 (38.88%) | 2,512 (23.2%) | 2,415 (22.3%) | 1,572 (14.52%) | 2,226 (20.56%) | 2,103 (19.42%) |
|  | 2021 | 13,241 | 45.35 (19.44) | 8,090 (61.1%) | 5,151 (38.9%) | 2,780 (21%) | 2,909 (21.97%) | 2,059 (15.55%) | 2,739 (20.69%) | 2,754 (20.8%) |
|  | 2022 | 10,082 | 46.56 (19.64) | 6,287 (62.36%) | 3,795 (37.64%) | 2,047 (20.3%) | 2,273 (22.55%) | 1,538 (15.25%) | 2,082 (20.65%) | 2,142 (21.25%) |
| Chronic obstructive pulmonary disease (COPD) | 2018 | 14361 | 67.44 (12.69) | 4,682 (32.6%) | 9,679 (67.4%) | 3,658 (25.47%) | 2,957 (20.59%) | 2,085 (14.52%) | 2,813 (19.59%) | 2,848 (19.83%) |
|  | 2019 | 14378 | 67.39 (12.71) | 4,845 (33.7%) | 9,533 (66.3%) | 3,568 (24.82%) | 2,933 (20.4%) | 2,029 (14.11%) | 2,890 (20.1%) | 2,958 (20.57%) |
|  | 2020 | 8439 | 67.87 (13.24) | 2,844 (33.7%) | 5,595 (66.3%) | 2,073 (24.56%) | 1,677 (19.87%) | 1,258 (14.91%) | 1,705 (20.2%) | 1,726 (20.45%) |
|  | 2021 | 10077 | 68.06 (13.18) | 3,619 (35.91%) | 6,458 (64.09%) | 2,432 (24.13%) | 2,106 (20.9%) | 1,480 (14.69%) | 1,948 (19.33%) | 2,111 (20.95%) |
|  | 2022 | 8507 | 67.62 (13.07) | 3,101 (36.45%) | 5,406 (63.55%) | 1,978 (23.25%) | 1,697 (19.95%) | 1,334 (15.68%) | 1,667 (19.6%) | 1,831 (21.52%) |
| Heart failure (HF) | 2018 | 12144 | 79.02 (11.21) | 6,527 (53.75%) | 5,617 (46.25%) | 3,078 (25.35%) | 2,661 (21.91%) | 1,886 (15.53%) | 2,300 (18.94%) | 2,219 (18.27%) |
|  | 2019 | 12869 | 79.16 (11.35) | 6,793 (52.79%) | 6,076 (47.21%) | 3,212 (24.96%) | 2,806 (21.8%) | 1,977 (15.36%) | 2,423 (18.83%) | 2,451 (19.05%) |
|  | 2020 | 11163 | 79.53 (11.26) | 5,916 (53%) | 5,247 (47%) | 2,847 (25.5%) | 2,306 (20.66%) | 1,667 (14.93%) | 2,123 (19.02%) | 2,220 (19.89%) |
|  | 2021 | 14701 | 79.14 (11.62) | 7,798 (53.04%) | 6,903 (46.96%) | 3,475 (23.64%) | 3,069 (20.88%) | 2,181 (14.84%) | 3,065 (20.85%) | 2,911 (19.8%) |
|  | 2022 | 11436 | 78.26 (12.15) | 5,816 (50.86%) | 5,620 (49.14%) | 2,816 (24.62%) | 2,283 (19.96%) | 1,791 (15.66%) | 2,230 (19.5%) | 2,316 (20.25%) |
| Hypercholesterolemia | 2018 | 27803 | 56.19 (13.56) | 15,635 (56.23%) | 12,168 (43.77%) | 6,571 (23.63%) | 6,276 (22.57%) | 4,336 (15.6%) | 5,488 (19.74%) | 5,132 (18.46%) |
|  | 2019 | 29773 | 56.2 (13.48) | 16,672 (56%) | 13,101 (44%) | 6,482 (21.77%) | 6,977 (23.43%) | 4,784 (16.07%) | 5,601 (18.81%) | 5,929 (19.91%) |
|  | 2020 | 21600 | 56.9 (13.66) | 12,509 (57.91%) | 9,091 (42.09%) | 5,235 (24.24%) | 4,869 (22.54%) | 3,387 (15.68%) | 4,069 (18.84%) | 4,040 (18.7%) |
|  | 2021 | 40806 | 58.1 (14) | 23,602 (57.84%) | 17,204 (42.16%) | 9,230 (22.62%) | 9,350 (22.91%) | 6,223 (15.25%) | 8,144 (19.96%) | 7,859 (19.26%) |
|  | 2022 | 30232 | 58.74 (14.15) | 17,263 (57.1%) | 12,969 (42.9%) | 6,660 (22.03%) | 6,745 (22.31%) | 5,019 (16.6%) | 5,987 (19.8%) | 5,821 (19.25%) |
| Hypertension | 2018 | 47643 | 60.18 (14.33) | 22,849 (47.96%) | 24,794 (52.04%) | 11,441 (24.01%) | 9,813 (20.6%) | 7,543 (15.83%) | 9,748 (20.46%) | 9,098 (19.1%) |
|  | 2019 | 48446 | 59.82 (14.24) | 23,209 (47.91%) | 25,237 (52.09%) | 11,757 (24.27%) | 9,849 (20.33%) | 7,631 (15.75%) | 9,917 (20.47%) | 9,292 (19.18%) |
|  | 2020 | 34482 | 60.2 (14.41) | 17,249 (50.02%) | 17,233 (49.98%) | 8,368 (24.27%) | 6,964 (20.2%) | 5,362 (15.55%) | 6,970 (20.21%) | 6,818 (19.77%) |
|  | 2021 | 54283 | 62.16 (14.61) | 27,053 (49.84%) | 27,230 (50.16%) | 12,221 (22.51%) | 11,286 (20.79%) | 8,280 (15.25%) | 11,282 (20.78%) | 11,214 (20.66%) |
|  | 2022 | 40083 | 62.78 (14.79) | 20,241 (50.5%) | 19,842 (49.5%) | 8,529 (21.28%) | 8,522 (21.26%) | 6,338 (15.81%) | 8,185 (20.42%) | 8,509 (21.23%) |
| Ischemic heart disease (IHD) | 2018 | 11178 | 68.26 (12.37) | 4,204 (37.61%) | 6,974 (62.39%) | 2,961 (26.49%) | 2,420 (21.65%) | 1,647 (14.73%) | 2,220 (19.86%) | 1,930 (17.27%) |
|  | 2019 | 11500 | 68.4 (12.15) | 4,388 (38.16%) | 7,112 (61.84%) | 3,020 (26.26%) | 2,571 (22.36%) | 1,625 (14.13%) | 2,202 (19.15%) | 2,082 (18.1%) |
|  | 2020 | 8035 | 68.45 (12.51) | 2,854 (35.52%) | 5,181 (64.48%) | 2,102 (26.16%) | 1,617 (20.12%) | 1,163 (14.47%) | 1,589 (19.78%) | 1,564 (19.46%) |
|  | 2021 | 10282 | 69.62 (12.44) | 3,915 (38.08%) | 6,367 (61.92%) | 2,391 (23.25%) | 2,286 (22.23%) | 1,658 (16.13%) | 1,957 (19.03%) | 1,990 (19.35%) |
|  | 2022 | 7217 | 69.34 (12.68) | 2,707 (37.51%) | 4,510 (62.49%) | 1,593 (22.07%) | 1,603 (22.21%) | 1,212 (16.79%) | 1,371 (19%) | 1,438 (19.93%) |
| Type 2 diabetes mellitus (T2DM) | 2018 | 27159 | 63.74 (13.8) | 11,820 (43.52%) | 15,339 (56.48%) | 6,722 (24.75%) | 4,875 (17.95%) | 4,258 (15.68%) | 5,554 (20.45%) | 5,750 (21.17%) |
|  | 2019 | 28705 | 63.45 (14) | 12,422 (43.27%) | 16,283 (56.73%) | 6,909 (24.07%) | 5,152 (17.95%) | 4,401 (15.33%) | 5,953 (20.74%) | 6,290 (21.91%) |
|  | 2020 | 19810 | 62.75 (14.05) | 8,651 (43.67%) | 11,159 (56.33%) | 5,057 (25.53%) | 3,490 (17.62%) | 2,829 (14.28%) | 4,119 (20.79%) | 4,315 (21.78%) |
|  | 2021 | 33811 | 64.22 (13.97) | 15,135 (44.76%) | 18,676 (55.24%) | 7,893 (23.34%) | 6,310 (18.66%) | 5,008 (14.81%) | 7,075 (20.93%) | 7,525 (22.26%) |
|  | 2022 | 24055 | 64.81 (14.36) | 10,982 (45.65%) | 13,073 (54.35%) | 5,515 (22.93%) | 4,545 (18.89%) | 3,676 (15.28%) | 4,952 (20.59%) | 5,367 (22.31%) |

### Figure S1. Age distribution of chronic diseases diagnoses during the study period

**
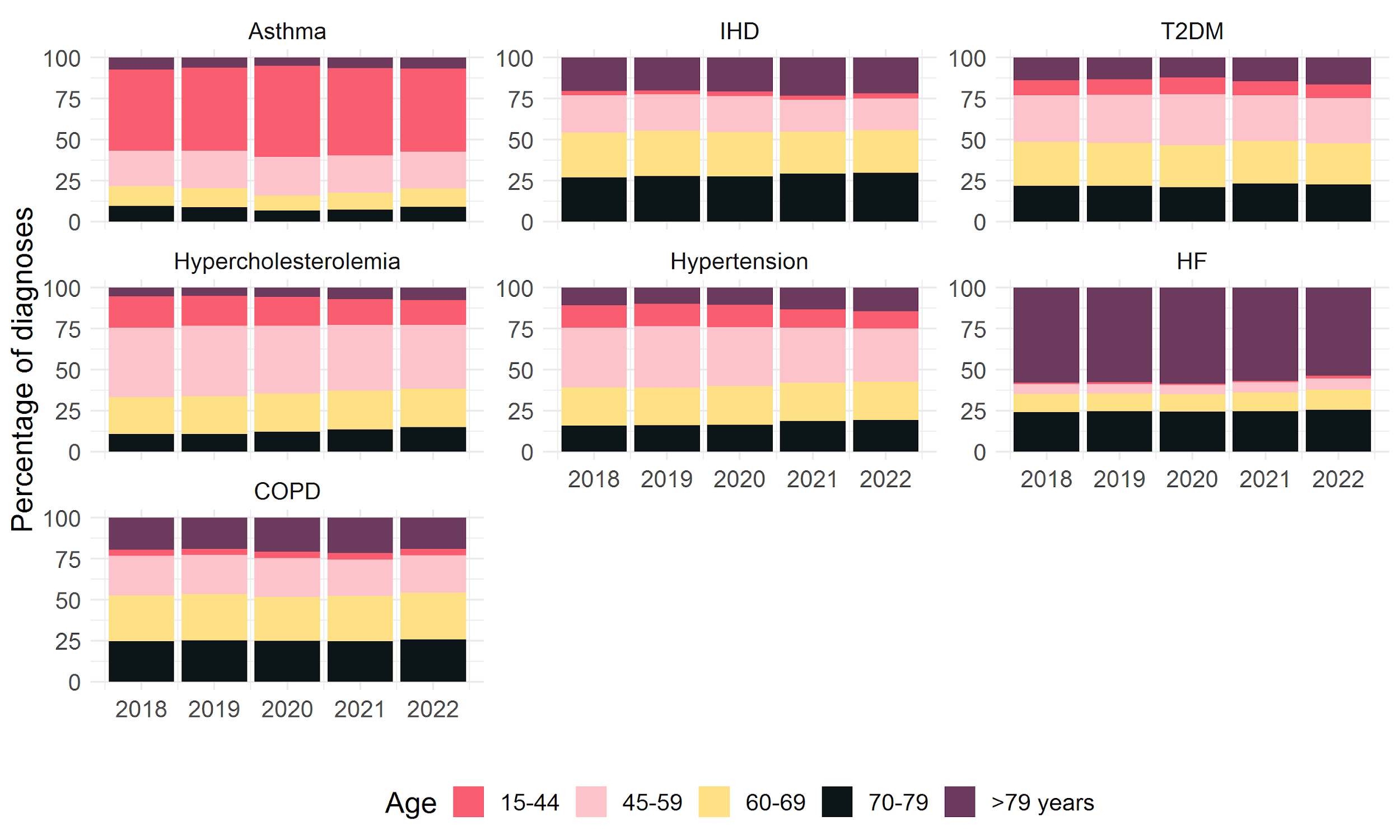
**

### Table S3. Coefficients of the segmented regression model

| **Chronic Disease** | **Coefficient** | **Beta** | **Lower 95% CI** | **Upper 95% CI** |
| --- | --- | --- | --- | --- |
| Asthma | Intercept (Beta_0) | 0.95414083 | 0.92767595 | 0.98060572 |
|  | beta_1 | -0.00014845 | -0.00020548 | -9.14E-05 |
|  | beta_2 | -0.37799784 | -0.41435526 | -0.34164042 |
|  | beta_3 | 0.00064171 | 0.00056718 | 0.00071624 |
| Chronic obstructive pulmonary disease (COPD) | Intercept (Beta_0) | 0.83558368 | 0.8129216 | 0.85824576 |
|  | beta_1 | -3.47E-05 | -8.35E-05 | 1.42E-05 |
|  | beta_2 | -0.53146105 | -0.56259417 | -0.50032792 |
|  | beta_3 | 0.00057448 | 0.00051066 | 0.0006383 |
| Heart failure (HF) | Intercept (Beta_0) | 0.73296189 | 0.71192532 | 0.75399847 |
|  | beta_1 | -5.61E-05 | -0.00010142 | -1.08E-05 |
|  | beta_2 | -0.16214575 | -0.19104576 | -0.13324573 |
|  | beta_3 | 0.00057531 | 0.00051607 | 0.00063456 |
| Hypercholesterolemia | Intercept (Beta_0) | 1.60684853 | 1.53868085 | 1.6750162 |
|  | beta_1 | 9.85E-05 | -4.84E-05 | 0.00024536 |
|  | beta_2 | -0.83045199 | -0.92410062 | -0.73680335 |
|  | beta_3 | 0.00218332 | 0.00199135 | 0.0023753 |
| Hypertension | Intercept (Beta_0) | 2.83727267 | 2.74354787 | 2.93099747 |
|  | beta_1 | -0.0002282 | -0.00043018 | -2.62E-05 |
|  | beta_2 | -1.22369037 | -1.35244933 | -1.0949314 |
|  | beta_3 | 0.00279009 | 0.00252613 | 0.00305404 |
| Ischemic heart disease (IHD) | Intercept (Beta_0) | 0.63937396 | 0.62377384 | 0.65497408 |
|  | beta_1 | -5.49E-06 | -3.91E-05 | 2.81E-05 |
|  | beta_2 | -0.26798453 | -0.28941595 | -0.24655311 |
|  | beta_3 | 0.00032898 | 0.00028505 | 0.00037292 |
| Type 2 diabetes mellitus (T2DM) | Intercept (Beta_0) | 1.59021683 | 1.53762557 | 1.64280809 |
|  | beta_1 | -2.87E-05 | -0.00014204 | 8.46E-05 |
|  | beta_2 | -0.70806023 | -0.78031001 | -0.63581045 |
|  | beta_3 | 0.00162458 | 0.00147647 | 0.00177269 |

### Figure S2 Incidence rate ratios (IRR) by year. Relative to year 2019

**IHD: Ischemic heart disease; T2DM: Type 2 diabetes mellitus; HF: Heart Failure; COPD: Chronic obstructive pulmonary disease**

**
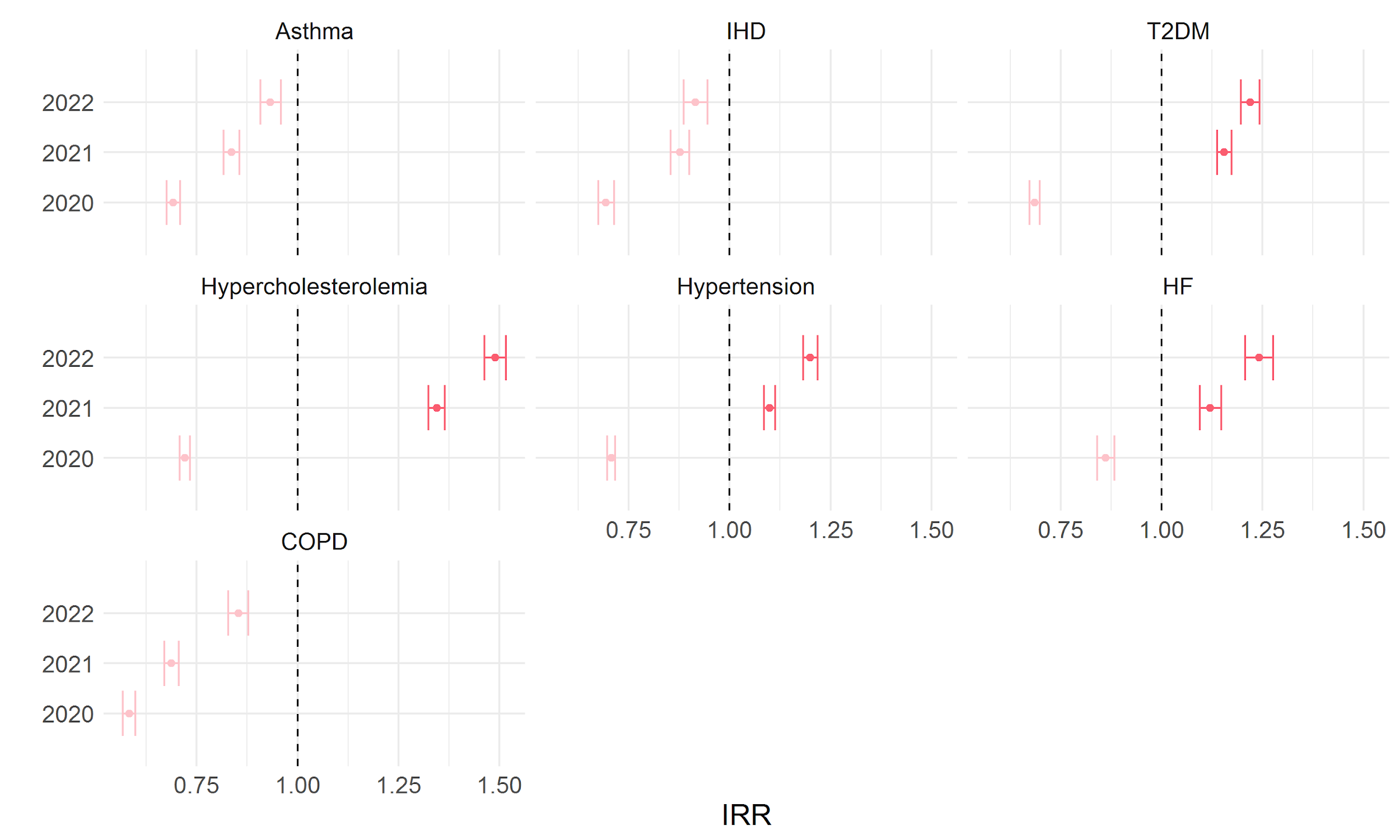
**

###

### Table S4. Observed and expected diagnoses during the pandemic period (from 14 March to 31 August 2022)

| **DIAGNOSTIC** | **Variable** | **Observed diagnoses (pandemic period)** | **Expected diagnoses for the pandemic period [IC95%]** | **Difference of diagnoses [IC95%]** | **Percetage of difference [IC95%]** |
| --- | --- | --- | --- | --- | --- |
| **Asthma** | **Total** | **30720** | **40439  [38245 to 42632]** | **-9719**  **[-11912 to -7525]** | **-24.03**  **[-27.94 to -19.68]** |
|  | 15-44 | 16472 | 19889  [18791 to 20986] | -3417  [-4514 to -2319] | -17.18  [-21.51 to -12.34] |
|  | 45-59 | 7021 | 9564  [8982 to 10146] | -2543  [-3125 to -1961] | -26.59  [-30.8 to -21.84] |
|  | 60-69 | 3086 | 4765  [4429 to 5100] | -1679  [-2014 to -1343] | -35.23  [-39.49 to -30.33] |
|  | 70-79 | 2322 | 3697  [3431 to 3964] | -1375  [-1642 to -1109] | -37.2  [-41.42 to -32.32] |
|  | >79 years | 1819 | 2466  [2281 to 2650] | -647  [-831 to -462] | -26.22  [-31.36 to -20.25] |
|  | Men | 11868 | 15606  [14711 to 16502] | -3738  [-4634 to -2843] | -23.95  [-28.08 to -19.33] |
|  | Women | 18852 | 24824  [23444 to 26203] | -5972  [-7351 to -4592] | -24.06  [-28.06 to -19.59] |
|  | Rural | 6578 | 9422  [8853 to 9990] | -2844  [-3412 to -2275] | -30.18  [-34.15 to -25.7] |
|  | 1st Q (least deprived) | 6884 | 8641  [8103 to 9180] | -1757  [-2296 to -1219] | -20.34  [-25.01 to -15.04] |
|  | 2nd Q | 4664 | 6031  [5645 to 6418] | -1367  [-1754 to -981] | -22.67  [-27.33 to -17.38] |
|  | 3rd Q | 6292 | 8259  [7760 to 8759] | -1967  [-2467 to -1468] | -23.82  [-28.16 to -18.92] |
|  | 4th Q (most deprived) | 6302 | 8102  [7598 to 8606] | -1800  [-2304 to -1296] | -22.22  [-26.77 to -17.06] |
| **Chronic obstructive pulmonary disease (COPD)** | **Total** | **23506** | **38150**  **[35814 to 40487]** | **-14644**  **[-16981 to -12308]** | **-38.39**  **[-41.94 to -34.37]** |
|  | 15-44 | 957 | 1360  [1229 to 1491] | -403  [-534 to -272] | -29.63  [-35.83 to -22.1] |
|  | 45-59 | 5294 | 9489  [8843 to 10136] | -4195  [-4842 to -3549] | -44.21  [-47.77 to -40.13] |
|  | 60-69 | 6473 | 11147  [10396 to 11898] | -4674  [-5425 to -3923] | -41.93  [-45.6 to -37.74] |
|  | 70-79 | 5891 | 9865  [9210 to 10521] | -3974  [-4630 to -3319] | -40.29  [-44 to -36.04] |
|  | >79 years | 4891 | 6946  [6506 to 7386] | -2055  [-2495 to -1615] | -29.59  [-33.78 to -24.83] |
|  | Men | 15119 | 25361  [23798 to 26924] | -10242  [-11805 to -8679] | -40.38  [-43.85 to -36.47] |
|  | Women | 8387 | 12817  [11971 to 13663] | -4430  [-5276 to -3584] | -34.56  [-38.61 to -29.94] |
|  | Rural | 5643 | 9488  [8863 to 10112] | -3845  [-4469 to -3220] | -40.52  [-44.19 to -36.33] |
|  | 1st Q (least deprived) | 4755 | 7788  [7262 to 8314] | -3033  [-3559 to -2507] | -38.95  [-42.81 to -34.52] |
|  | 2nd Q | 3551 | 5437  [5042 to 5831] | -1886  [-2280 to -1491] | -34.68  [-39.1 to -29.57] |
|  | 3rd Q | 4608 | 7659  [7130 to 8189] | -3051  [-3581 to -2522] | -39.84  [-43.73 to -35.37] |
|  | 4th Q (most deprived) | 4949 | 7803  [7271 to 8336] | -2854  [-3387 to -2322] | -36.58  [-40.63 to -31.94] |
| **Heart failure (HF)** | **Total** | **34461** | **33496**  **[31686 to 35306]** | **965**  **[-845 to 2775]** | **2.88**  **[-2.39 to 8.76]** |
|  | 15-44 | 363 | 305  [253 to 357] | 58  [6 to 110] | 19.05  [1.68 to 43.58] |
|  | 45-59 | 2196 | 1967  [1800 to 2133] | 229  [63 to 396] | 11.66  [2.93 to 22] |
|  | 60-69 | 3958 | 3706  [3445 to 3966] | 252  [-8 to 513] | 6.81  [-0.2 to 14.89] |
|  | 70-79 | 8532 | 8580  [8043 to 9118] | -48  [-586 to 489] | -0.56  [-6.43 to 6.09] |
|  | >79 years | 19412 | 18755  [17730 to 19780] | 657  [-368 to 1682] | 3.5  [-1.86 to 9.49] |
|  | Men | 16453 | 15796  [14906 to 16685] | 657  [-232 to 1547] | 4.16  [-1.39 to 10.38] |
|  | Women | 18008 | 17703  [16707 to 18699] | 305  [-691 to 1301] | 1.72  [-3.69 to 7.79] |
|  | Rural | 8430 | 8439  [7940 to 8937] | -9  [-507 to 490] | -0.1  [-5.68 to 6.17] |
|  | 1st Q (least deprived) | 7047 | 7278  [6830 to 7727] | -231  [-680 to 217] | -3.18  [-8.8 to 3.18] |
|  | 2nd Q | 5238 | 5072  [4736 to 5408] | 166  [-170 to 502] | 3.27  [-3.15 to 10.59] |
|  | 3rd Q | 6877 | 6304  [5895 to 6712] | 573  [165 to 982] | 9.1  [2.46 to 16.66] |
|  | 4th Q (most deprived) | 6869 | 6426  [6020 to 6832] | 443  [37 to 849] | 6.9  [0.55 to 14.11] |
| **Hypercholesterolemia** | **Total** | **85456** | **78755**  **[73575 to 83936]** | **6701**  **[1520 to 11881]** | **8.51**  **[1.81 to 16.15]** |
|  | 15-44 | 13214 | 14249  [13257 to 15242] | -1035  [-2028 to -43] | -7.27  [-13.3 to -0.32] |
|  | 45-59 | 34055 | 34842  [32515 to 37169] | -787  [-3114 to 1540] | -2.26  [-8.38 to 4.74] |
|  | 60-69 | 20149 | 18660  [17399 to 19921] | 1489  [228 to 2750] | 7.98  [1.15 to 15.8] |
|  | 70-79 | 11950 | 8706  [8078 to 9335] | 3244  [2615 to 3872] | 37.25  [28.01 to 47.94] |
|  | >79 years | 6088 | 3788  [3478 to 4099] | 2300  [1989 to 2610] | 60.7  [48.54 to 75.03] |
|  | Men | 36020 | 34901  [32564 to 37238] | 1119  [-1218 to 3456] | 3.21  [-3.27 to 10.61] |
|  | Women | 49436 | 43853  [40943 to 46762] | 5583  [2674 to 8493] | 12.73  [5.72 to 20.74] |
|  | Rural | 19625 | 17176  [16021 to 18332] | 2449  [1293 to 3604] | 14.26  [7.06 to 22.5] |
|  | 1st Q (least deprived) | 19244 | 18514  [17232 to 19796] | 730  [-552 to 2012] | 3.94  [-2.79 to 11.68] |
|  | 2nd Q | 13473 | 12666  [11782 to 13549] | 807  [-76 to 1691] | 6.37  [-0.56 to 14.35] |
|  | 3rd Q | 16784 | 14907  [13852 to 15962] | 1877  [822 to 2932] | 12.59  [5.15 to 21.17] |
|  | 4th Q (most deprived) | 16330 | 15519  [14452 to 16585] | 811  [-255 to 1878] | 5.23  [-1.54 to 12.99] |
| **Hypertension** | **Total** | **117199** | **128125**  **[120757 to 135493]** | **-10926**  **[-18294 to -3558]** | **-8.53**  **[-13.5 to -2.95]** |
|  | 15-44 | 13366 | 17080  [16060 to 18100] | -3714  [-4734 to -2694] | -21.74  [-26.16 to -16.77] |
|  | 45-59 | 39250 | 49740  [46793 to 52688] | -10490  [-13438 to -7543] | -21.09  [-25.5 to -16.12] |
|  | 60-69 | 27399 | 30561  [28683 to 32438] | -3162  [-5039 to -1284] | -10.35  [-15.54 to -4.48] |
|  | 70-79 | 21606 | 21143  [19846 to 22439] | 463  [-833 to 1760] | 2.19  [-3.71 to 8.87] |
|  | >79 years | 15578 | 11954  [11273 to 12635] | 3624  [2943 to 4305] | 30.32  [23.29 to 38.18] |
|  | Men | 58126 | 67099  [63134 to 71064] | -8973  [-12938 to -5008] | -13.37  [-18.21 to -7.93] |
|  | Women | 59073 | 61057  [57575 to 64540] | -1984  [-5467 to 1498] | -3.25  [-8.47 to 2.6] |
|  | Rural | 26375 | 31208  [29339 to 33077] | -4833  [-6702 to -2964] | -15.49  [-20.26 to -10.1] |
|  | 1st Q (least deprived) | 24446 | 25926  [24419 to 27433] | -1480  [-2987 to 27] | -5.71  [-10.89 to 0.11] |
|  | 2nd Q | 18093 | 20298  [19066 to 21529] | -2205  [-3436 to -973] | -10.86  [-15.96 to -5.1] |
|  | 3rd Q | 24082 | 26091  [24546 to 27636] | -2009  [-3554 to -464] | -7.7  [-12.86 to -1.89] |
|  | 4th Q (most deprived) | 24203 | 24684  [23189 to 26178] | -481  [-1975 to 1014] | -1.95  [-7.55 to 4.37] |
| **Ischemic heart disease (IHD)** | **Total** | **23209** | **29473**  **[27751 to 31196]** | **-6264**  **[-7987 to -4542]** | **-21.25**  **[-25.6 to -16.37]** |
|  | 15-44 | 643 | 680  [600 to 760] | -37  [-117 to 43] | -5.46  [-15.43 to 7.17] |
|  | 45-59 | 4641 | 6729  [6293 to 7165] | -2088  [-2524 to -1652] | -31.03  [-35.23 to -26.25] |
|  | 60-69 | 6038 | 8452  [7911 to 8992] | -2414  [-2954 to -1873] | -28.56  [-32.85 to -23.68] |
|  | 70-79 | 6724 | 8379  [7818 to 8940] | -1655  [-2216 to -1094] | -19.75  [-24.78 to -13.99] |
|  | >79 years | 5163 | 5734  [5337 to 6131] | -571  [-968 to -174] | -9.96  [-15.79 to -3.26] |
|  | Men | 14593 | 18321  [17244 to 19397] | -3728  [-4804 to -2651] | -20.35  [-24.77 to -15.37] |
|  | Women | 8616 | 11170  [10463 to 11877] | -2554  [-3261 to -1847] | -22.86  [-27.45 to -17.65] |
|  | Rural | 5460 | 7847  [7315 to 8380] | -2387  [-2920 to -1855] | -30.42  [-34.85 to -25.36] |
|  | 1st Q (least deprived) | 5040 | 6468  [6014 to 6921] | -1428  [-1881 to -974] | -22.07  [-27.18 to -16.19] |
|  | 2nd Q | 3696 | 4185  [3872 to 4498] | -489  [-802 to -176] | -11.68  [-17.83 to -4.54] |
|  | 3rd Q | 4447 | 5680  [5283 to 6076] | -1233  [-1629 to -836] | -21.71  [-26.81 to -15.83] |
|  | 4th Q (most deprived) | 4566 | 5320  [4959 to 5680] | -754  [-1114 to -393] | -14.17  [-19.62 to -7.92] |
| **Type 2 diabetes mellitus (T2DM)** | **Total** | **71506** | **74341**  **[69791 to 78892]** | **-2835**  **[-7386 to 1715]** | **-3.81**  **[-9.36 to 2.46]** |
|  | 15-44 | 6301 | 6756  [6306 to 7207] | -455  [-906 to -5] | -6.74  [-12.57 to -0.08] |
|  | 45-59 | 20401 | 22537  [21113 to 23960] | -2136  [-3559 to -712] | -9.48  [-14.85 to -3.37] |
|  | 60-69 | 18189 | 20114  [18826 to 21403] | -1925  [-3214 to -637] | -9.57  [-15.01 to -3.38] |
|  | 70-79 | 16138 | 16762  [15652 to 17872] | -624  [-1734 to 486] | -3.72  [-9.7 to 3.1] |
|  | >79 years | 10477 | 9497  [8875 to 10120] | 980  [357 to 1602] | 10.32  [3.53 to 18.05] |
|  | Men | 39372 | 42326  [39707 to 44945] | -2954  [-5573 to -335] | -6.98  [-12.4 to -0.84] |
|  | Women | 32134 | 32045  [30037 to 34053] | 89  [-1919 to 2097] | 0.28  [-5.63 to 6.98] |
|  | Rural | 16963 | 18101  [16957 to 19246] | -1138  [-2283 to 6] | -6.29  [-11.86 to 0.04] |
|  | 1st Q (least deprived) | 13245 | 13313  [12424 to 14202] | -68  [-957 to 821] | -0.51  [-6.74 to 6.61] |
|  | 2nd Q | 10625 | 11281  [10533 to 12029] | -656  [-1404 to 92] | -5.82  [-11.67 to 0.87] |
|  | 3rd Q | 14781 | 15553  [14535 to 16571] | -772  [-1790 to 246] | -4.96  [-10.8 to 1.69] |
|  | 4th Q (most deprived) | 15892 | 16129  [15088 to 17169] | -237  [-1277 to 804] | -1.47  [-7.44 to 5.33] |

### Table S5. Date of compensation and excess of diagnoses for each chronic disease. Total and stratified by age groups, sex and socioeconomic status

| **Chronic disease** | **Recovery/Excess** | **Variable** | **Date** |
| --- | --- | --- | --- |
| Heart failure (HF) | Recovery | Total | 16/12/2021 |
| Heart failure (HF) | Recovery | 15-44 | 23/09/2021 |
| Heart failure (HF) | Recovery | 45-59 | 06/10/2021 |
| Heart failure (HF) | Recovery | 60-69 | 17/11/2021 |
| Heart failure (HF) | Recovery | 70-79 | 25/02/2022 |
| Heart failure (HF) | Recovery | >79 years | 17/06/2021 |
| Heart failure (HF) | Recovery | Men | 21/12/2021 |
| Heart failure (HF) | Recovery | Women | 23/11/2021 |
| Heart failure (HF) | Recovery | Rural | 17/02/2022 |
| Heart failure (HF) | Recovery | 1st Q (least deprived) | 25/03/2022 |
| Heart failure (HF) | Recovery | 2nd Q | 03/12/2021 |
| Heart failure (HF) | Recovery | 3rd Q | 02/06/2021 |
| Heart failure (HF) | Recovery | 4th Q (most deprived) | 11/06/2021 |
| Heart failure (HF) | Excess | 15-44 | 27/06/2022 |
| Heart failure (HF) | Excess | 45-59 | 21/04/2022 |
| Heart failure (HF) | Excess | 60-69 | 16/06/2022 |
| Heart failure (HF) | Excess | 3rd Q | 18/03/2022 |
| Heart failure (HF) | Excess | 4th Q (most deprived) | 01/06/2022 |
| Hypercholesterolemia | Recovery | Total | 05/11/2021 |
| Hypercholesterolemia | Recovery | 15-44 | 25/05/2022 |
| Hypercholesterolemia | Recovery | 45-59 | 24/03/2022 |
| Hypercholesterolemia | Recovery | 60-69 | 29/10/2021 |
| Hypercholesterolemia | Recovery | 70-79 | 17/06/2021 |
| Hypercholesterolemia | Recovery | >79 years | 07/06/2021 |
| Hypercholesterolemia | Recovery | Men | 11/02/2022 |
| Hypercholesterolemia | Recovery | Women | 23/09/2021 |
| Hypercholesterolemia | Recovery | Rural | 15/06/2021 |
| Hypercholesterolemia | Recovery | 1st Q (least deprived) | 02/12/2021 |
| Hypercholesterolemia | Recovery | 2nd Q | 28/01/2022 |
| Hypercholesterolemia | Recovery | 3rd Q | 06/10/2021 |
| Hypercholesterolemia | Recovery | 4th Q (most deprived) | 16/12/2021 |
| Hypercholesterolemia | Excess | Total | 26/05/2022 |
| Hypercholesterolemia | Excess | 60-69 | 01/06/2022 |
| Hypercholesterolemia | Excess | 70-79 | 20/09/2021 |
| Hypercholesterolemia | Excess | >79 years | 03/08/2021 |
| Hypercholesterolemia | Excess | Women | 24/03/2022 |
| Hypercholesterolemia | Excess | Rural | 25/02/2022 |
| Hypercholesterolemia | Excess | 3rd Q | 07/04/2022 |
| Hypertension | Recovery | 70-79 | 17/02/2022 |
| Hypertension | Recovery | >79 years | 07/08/2021 |
| Hypertension | Recovery | Women | 08/04/2022 |
| Hypertension | Recovery | 4th Q (most deprived) | 29/03/2022 |
| Hypertension | Excess | >79 years | 04/11/2021 |
| Ischemic heart disease (IHD) | Recovery | 15-44 | 30/09/2021 |
| Type 2 diabetes mellitus (T2DM) | Recovery | Total | 28/04/2022 |
| Type 2 diabetes mellitus (T2DM) | Recovery | 15-44 | 24/05/2022 |
| Type 2 diabetes mellitus (T2DM) | Recovery | 70-79 | 13/04/2022 |
| Type 2 diabetes mellitus (T2DM) | Recovery | >79 years | 15/12/2021 |
| Type 2 diabetes mellitus (T2DM) | Recovery | Women | 18/02/2022 |
| Type 2 diabetes mellitus (T2DM) | Recovery | Rural | 01/06/2022 |
| Type 2 diabetes mellitus (T2DM) | Recovery | 1st Q (least deprived) | 22/02/2022 |
| Type 2 diabetes mellitus (T2DM) | Recovery | 2nd Q | 03/06/2022 |
| Type 2 diabetes mellitus (T2DM) | Recovery | 3rd Q | 11/05/2022 |
| Type 2 diabetes mellitus (T2DM) | Recovery | 4th Q (most deprived) | 09/03/2022 |
| Type 2 diabetes mellitus (T2DM) | Excess | >79 years | 20/05/2022 |
